# Reanalysis of the association between reduction in long-term PM_2.5_ concentrations and improved life expectancy

**DOI:** 10.1186/s12940-021-00785-0

**Published:** 2021-09-13

**Authors:** Sun-Young Kim, Arden C. Pope, Julian D. Marshall, Neal Fann, Lianne Sheppard

**Affiliations:** 1 Department of Cancer Control and Population Health, Graduate School of Cancer Science and Policy, National Cancer Center, Goyang, Gyeonggi Korea; 2 Department of Environmental and Occupational Health Sciences, University of Washington, Seattle, WA USA; 3 Department of Economics, Brigham Young University, Provo, UT USA; 4 Department of Civil and Environmental Engineering, University of Washington, Seattle, WA USA; 5 Office of Air Quality, Planning and Standards, US Environmental Protection Agency, RTP, Durham, NC USA; 6 Department of Biostatistics, University of Washington, Seattle, WA USA

**Keywords:** Fine particulate matter, Life expectancy, Long-term exposure, Measured exposure, Modeled exposure, Prediction

## Abstract

**Background:**

Much of the current evidence of associations between long-term PM_2.5_ and health outcomes relies on national or regional analyses using exposures derived directly from regulatory monitoring data. These findings could be affected by limited spatial coverage of monitoring data, particularly for time periods before spatially extensive monitoring began in the late 1990s. For instance, Pope et al. (2009) showed that between 1980 and 2000 a 10 μg/m^3^ reduction in PM_2.5_ was associated with an average 0.61 year (standard error (SE) = 0.20) longer life expectancy. That analysis used 1979–1983 averages of PM_2.5_ across 51 U.S. Metropolitan Statistical Areas (MSAs) computed from about 130 monitoring sites. Our reanalysis re-examines this association using modeled PM_2.5_ in order to assess population- or spatially-representative exposure. We hypothesized that modeled PM_2.5_ with finer spatial resolution provides more accurate health effect estimates compared to limited monitoring data.

**Methods:**

We used the same data for life expectancy and confounders, as well as the same analysis models, and investigated the same 211 continental U.S. counties, as Pope et al. (2009). For modeled PM_2.5_, we relied on a previously-developed point prediction model based on regulatory monitoring data for 1999–2015 and back-extrapolation to 1979. Using this model, we predicted annual average concentrations at centroids of all 72,271 census tracts and 12,501 25-km national grid cells covering the contiguous U.S., to represent population and space, respectively. We averaged these predictions to the county for the two time periods (1979–1983 and 1999–2000), whereas the original analysis used MSA averages given limited monitoring data. Finally, we estimated regression coefficients for PM_2.5_ reduction on life expectancy improvement over the two periods, adjusting for area-level confounders.

**Results:**

A 10 μg/m^3^ decrease in modeled PM_2.5_ based on census tract and national grid predictions was associated with 0.69 (standard error (SE) = 0.31) and 0.81 (0.29) -year increases in life expectancy. These estimates are higher than the estimate of Pope et al. (2009); they also have larger SEs likely because of smaller variability in exposure predictions, a standard property of regression. Two sets of effect estimates, however, had overlapping confidence intervals.

**Conclusions:**

Our approach for estimating population- and spatially-representative PM_2.5_ concentrations based on census tract and national grid predictions, respectively, provided generally consistent findings to the original findings using limited monitoring data. This finding lends additional support to the evidence that reduced fine particulate matter contributes to extended life expectancy.

**Supplementary Information:**

The online version contains supplementary material available at 10.1186/s12940-021-00785-0.

## Background

Evidence of the association between long-term exposure to particles less than or equal to 2.5 µm in diameter (PM_2.5_) and adverse health outcomes, first reported in early 1990s [1, 2], has continued to grow [3–9]. Many of these epidemiological studies were conducted in the U.S., Canada, and Europe, and relied on regulatory monitoring data; however, nationwide and population-focused regulatory monitoring for PM_2.5_ did not begin until 1999. Lack of available monitoring data affects the time period available to investigate the health effects of PM_2.5_. The influence could be particularly large for studies evaluating the impact of changing PM_2.5_ on health over several decades.

The limited historical monitoring data for PM_2.5_ is more challenging when research considers concentration variability in both space and time. As an example of an effort that was restricted to regions with available monitoring data over a long time period, Pope et al. (2009) investigated the association between the change in PM_2.5_ from 1980s through 2000s and the change in life expectancy. They concluded that improved life expectancy was attributed to decreased PM_2.5_ concentrations [10]. This 20-year change in PM_2.5_ was characterized directly from regulatory monitoring data. For the 2000s, the data came from approximately 1,000 sites in more than 900 major metropolitan areas over the continental U.S. in the Federal Reference Methods (FRM) network [11]. However, for the 1980s they relied on measurements from the Inhalable Particulate Network (IPN) at only about 130 sites which were located in 51 major metropolitan areas [12, 13]. Given these limited monitoring data, PM_2.5_ exposure was aggregated to the Metropolitan Statistical Area (MSA) and the analysis was restricted to 211 out of more than 3,000 counties. While many cohort studies have improved spatial coverage by estimating exposure at people’s residences from air pollution prediction models, they have been unable to develop predictions in the 1980’s. For instance, a recent study expanded Pope et al.’s analysis to all U.S. counties by using predicted PM_2.5_ concentrations; however, because of the unavailability of spatially extensive monitoring data before 1999, they focused on the change during 1999–2015 [14].

A historical PM_2.5_ prediction model such as the one we previously developed can help overcome this space–time limitation. Our pointwise spatio-temporal prediction model allows estimation of annual average concentrations of PM_2.5_ at arbitrary point locations in the continental U.S. for 1980–2010; this temporal range includes the years when extensive spatial monitoring data are unavailable [15]. In external validation with PM_2.5_ data measured before 1999, from the Interagency Monitoring of Protected Visual Environments (IMPROVE) network and the Southern California Children’s Health Study, the model generally performed well, with R^2^ values over 0.7. These modeled PM_2.5_ exposures over the 30-year period can provide representative exposure estimates, and thus advance our understanding of the effectiveness of PM_2.5_ reduction for human health.

Using population-representative exposure estimates from the historical PM_2.5_ prediction model, we aimed to re-examine the association between PM_2.5_ reduction and life expectancy increase for 1980–2000. Our hypothesis is that modeled PM_2.5_ in 1980s at finer spatial resolution provides more accurate estimates of the association with changes in life expectancy compared to limited PM_2.5_ measurements. Specifically, we replaced PM_2.5_ MSA averages (which were derived from limited monitoring data) in Pope et al. (2009) with modeled population- and spatially-representative county-average exposure while maintaining the same data for life expectancy and covariates, as well as the health analysis models used in the original analysis. Then, we compared the estimates of association with life expectancy between the new and the original findings.

## Methods

### Historical prediction model for PM_2.5_

The historical prediction model uses the same PM_2.5_ prediction model framework as in the Multiethnic Study of Atherosclerosis and Air Pollution (MESA Air) [16–19]. These MESA Air exposure model predicted 2-week average concentrations at any location in six U.S. metropolitan cities, using monitoring data from two PM_2.5_ regulatory monitoring networks, the FRM and IMPROVE, and a cohort-focused monitoring campaign. In contrast, the historical prediction model relied only on regulatory monitoring data for 1999–2010 and predicted annual average concentrations in the continental U.S. between 1980 and 2010 including the period before spatially extensive monitoring for PM_2.5_ began. For this paper, we extended the extrapolation to 1979. Details in the input data and modeling procedure were described elsewhere [15]. In brief, this model consists of three components to characterize temporal and spatial patterns of annual average concentrations of PM_2.5_: a spatially-varying long-term mean, a spatially-varying temporal trend, and temporally-independent and spatially-dependent spatio-temporal residuals. We estimated the single temporal trend, which approximates linearity, using the data for 1999–2010. Then, to estimate the temporal trend before 1999 we explored various data sources using emissions, meteorology, visibility, and PM_2.5_ sulfate: our findings suggested that the back-extrapolated trend was also linear. Based on these extensive exploratory analyses, we extrapolated the linear trend to the period prior to 1999. The temporal trend was scaled by a spatially-varying trend coefficient to reflect spatial heterogeneity of the temporal trend, as identified in monitor levels. We characterized the spatially varying long-term mean and trend in a universal kriging framework with dimension-reduced summary predictors. These summary predictors were estimated from more than 800 geographic variables using partial least squares (PLS). These geographic variables computed based on distances and multiple buffers characterize potential pollution sources such as traffic, land use, population, emissions, and vegetation. PLS finds the linear combination of geographic variables which is most correlated with PM_2.5_ annual averages [20].

### County-average PM_2.5 _estimation

Using the pointwise historical prediction model, we estimated PM_2.5_ annual average concentrations in 1979–1983 and 1999–2000 at 1) 72,271 census tract centroids based on the year 2010 census and 2) 12,501 national grid coordinates that cover the contiguous lower 48 states. We obtained census tract boundaries from the National Historical Geographic Information System (www.nhgis.org), and calculated the centroid, as the geometric center, for each of the census tracts using ArcGIS 10.2 Geographic Information System software (Additional file 1: Figure S1). The 12,501 grid coordinates were obtained from a 25-km grid in the continental U.S. Pope et al. (2009) used the PM_2.5_ concentrations averaged to 51 MSAs for the health analysis of life expectancy estimates in 211 counties. Pope and coauthors computed average PM_2.5_ concentrations for 1979–1983 with available data measured at about 130 IPN sites [7], and at more than 500 FRM sites for 1999–2000 [10] (“measured” PM_2.5_). Using our predicted PM_2.5_ concentrations at 72,271 census tract centroids and 12,501 national grid coordinates, we averaged PM_2.5_ to the same 211 counties to be directly comparable with the spatial scale of Pope et al. (2009) life expectancy estimates for our primary analysis (“modeled” PM_2.5_). As census tract centroids represent populated locations while national grid coordinates represent spatially evenly distributed locations, we treated the county-level averages of those predictions as population- and spatially-representative concentrations, respectively. Then, we also computed the averages across the 51 MSAs to be directly comparable with the spatial scale of their exposure estimates for a sensitivity analysis. We averaged predictions across 4 to 2,343 (median = 56) and 6 to 2,343 (372) census tract centroids to obtain 211 county and 51 MSA averages, respectively.

### Life expectancy reanalysis

To compare against results from Pope et al. (2009), we examined the association between PM_2.5_ reduction and life expectancy increase for 1980–2000 using the same data for life expectancy and covariates in 211 U.S. counties (Additional file 1: Figure S2), and the same seven health analysis models. The only difference from the original analysis was the replacement of MSA-average PM_2.5_ exposure based on IPN and FRM measurements with our county-average historical predictions. The life expectancy estimates were for two five-year periods (1978–1982 and 1997–2001) and were estimated using mortality statistics from the National Center for Health Statistics and population from the U.S. Census. The detailed procedure for the computation of annual-average life expectancy and area-level characteristics has been described elsewhere [21].

Using multiple linear regression with the new exposure predictions, we assessed the association between PM_2.5_ and life expectancy in each time period separately, and for differences between the two periods. We estimated robust standard errors by clustering on MSA. The seven confounder models included progressively expanded sets of county-level socio-demographic variables, proxy variables for smoking prevalence, and smaller subsets of counties as we describe here. Whereas Model 1 only included PM_2.5_, Models 2 and 3 additionally adjusted for progressively expanded sets of confounders. While Model 2 consisted of average income, total population, and proportions of 5-yr in-migration, high-school graduates, urban residence, black population, and Hispanic population, Model 3 also added two surrogate variables for smoking: mortality rates for lung cancer and chronic obstructive pulmonary disease (COPD). Model 4, as the primary model in our analysis as well as in Pope et al. 2009, included income, population, proportion of black population, and mortality rates for lung cancer and COPD. In addition, confounders used in Models 3 and 4 were applied to the restricted study area with high population. Out of the 211 counties included in the primary analysis, we applied Model 4 to 127 counties with population greater than 100 thousand in 1986 (Model 5) and Models 3 and 4 to the 51 counties that had the largest population in each MSA (Models 6 and 7).

### Sensitivity analysis

We performed two sensitivity analyses to test the robustness of our results to address the role of spatially-refined exposure and exposure averaging period, respectively. First, we used MSA-average estimates of our census tract or national grid historical predictions instead of county-average estimates. Second, we investigated different exposure time periods, using one or multiple years of PM_2.5_ averages in the two five-year time periods (1979–1983 and 1999–2004) and compared these to our main findings that were based on the same averaging periods as Pope et al. 2009’s (1979–1983 and 1999–2000). This analysis examined whether our findings were affected by the number of years included in the exposure averages or the selection of specific years in each period.

## Results

### Measured and modeled PM_2.5_

Modeled county- and MSA-average PM_2.5_ estimated by the historical prediction model tended to be lower than measured MSA-averages derived by PM_2.5_ regulatory monitoring data in both periods. The average concentration of census tract predictions was 17.85 μg/m^3^ in 1979–1983 and 12.90 μg/m^3^ in 1999–2000, compared to 20.62 and 14.10 μg/m^3^ for the measurements; national grid predictions were slightly lower than census tract predictions, with the average concentration of 16.29 and 11.95 μg/m^3^ in 1979–1983 and 1999–2000, respectively (Table 1). While variability was also lower in modeled PM_2.5_ than measured PM_2.5_ in 1979–1983, the more recent 1999–2000 period gave similar variability. Despite the consistent means between MSA and county averages in predictions, variability was slightly larger for county averages than MSA averages. For census tract and national grid predictions, national grid predictions, many of which were estimated at non-residential locations, were more variable than census tract predictions.

**Table 1:** Summary statistics of average PM_2.5_ concentrations (μg/m^3^) in 51 Metropolitan Statistical Areas (MSAs) based on measurements from regulatory monitoring networks (Inhalable Particulate Network (IPN) or Federal Reference Method (FRM)), and in 211 counties or 51 MSAs based on estimates by the historical prediction model for 1979–1983 and 1999–2000

Period	Exposure assessment	PM_2.5_				
		Min	Median	Max	Mean^c^	SD
1979–1983	Measurement (IPN)^a^: MSA average	10.77	20.81	30.01	20.62	4.36
	Census tract prediction: MSA average	10.61	18.46	24.84	17.85	2.42
	Census tract prediction: county average	9.73	18.09	24.84	17.85	2.89
	National grid prediction: MSA average	5.14	17.12	23.00	16.29	3.32
	National grid prediction: county average	3.66	16.92	24.77	16.29	3.75
1999–2000	Measurement (FRM)^b^: MSA average	5.80	14.50	20.20	14.10	2.86
	Census tract prediction: MSA average	5.90	13.51	19.77	12.90	2.65
	Census tract prediction: county average	5.54	12.94	19.77	12.90	2.82
	National grid prediction: MSA average	3.00	12.79	16.93	11.95	3.11
	National grid prediction: county average	2.32	12.36	19.62	11.95	3.28

^a^ Inhalable Particulate Network

^b^ Federal Reference Method

^c^ MSA and county averages appear the same due to rounding; 1979–1983 IPN values differ slightly from Pope et al. (2009) due to rounding

**Fig. 1:**
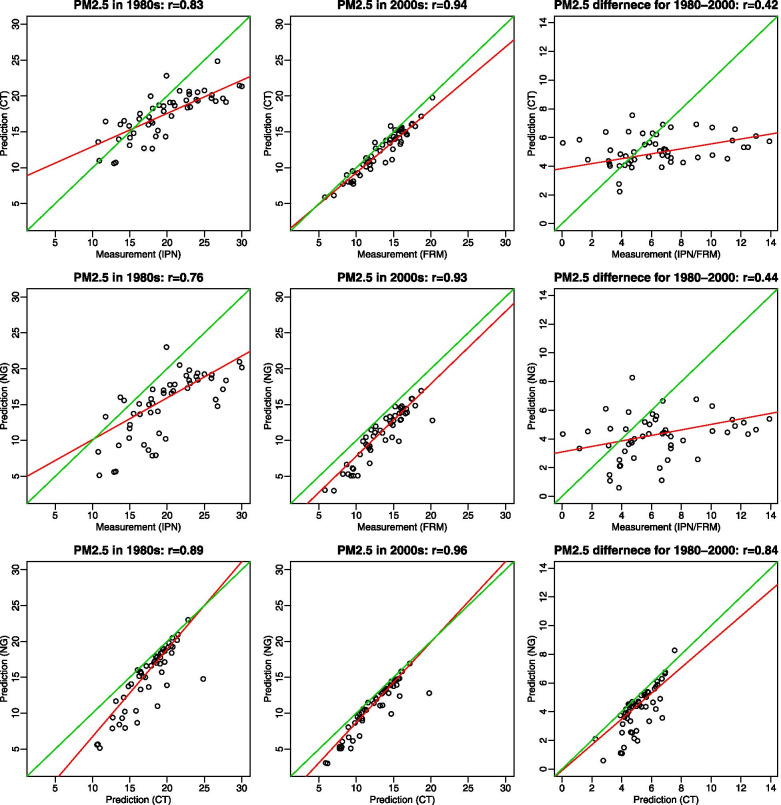
Scatter plots of PM_2.5_ concentrations based on measurements from regulatory monitoring networks (Inhalable Particulate Network (IPN) or Federal Reference Method (FRM)) and predictions at census tract centroids and national grid coordinates estimated by the historical prediction model across 51 Metropolitan Statistical Areas by 1980s (1979–1983), 2000s (1999–2000), and 1980s-2000s (1979–1983 minus 1999–2000) (green and red lines for the identity and best-fitted lines, respectively; the 1^st^ to 3^rd^ row displaying measured vs. census tract-based modeled PM_2.5_, measured vs. national grid-based modeled PM_2.5_, and modeled census tract-based vs. modeled national grid-based PM_2.5_, respectively)

Across 51 MSAs, measured versus modeled PM_2.5_ tended to be more dissimilar as concentration increased. Figure 1 shows MSA-average concentrations of modeled PM_2.5_ compared to measured PM_2.5_ in each of the two periods and their differences between the periods. Correlation between MSA averages of modeled and measured PM_2.5_ was lower in the early period for 1979–1983 (Pearson correlation coefficient = 0.76–0.83) than in the recent period for 1999–2000 (0.93–0.94) (Fig. 1). The difference between the two periods gave poor correlation (0.42–0.44), although both PM_2.5_ showed a decreasing trend between the two time periods. When county-average predictions were compared to MSA measurements, the pattern of lower correlation in the early period than in the recent period was consistent, with lower correlation coefficients (Pearson correlation coefficient = 0.68–0.70 and 0.88–0.89 for 1979–1983 and 1999–2000, respectively) than those for MSA-average predictions (Additional file 1: Figure S3-S4). Prediction maps of county-average PM_2.5_ in 1980, 1990, 2000, and 2010 based on census tract predictions also showed the overall decreasing trend in most counties for 1980–2010 (Additional file 1: Figure S5). The concentrations were higher in the eastern region and California’s Central Valley, and the reduction over time was also large in these areas.

The census tract and national grid PM_2.5_ predictions were highly correlated in both time periods (Pearson correlation coefficient = 0.89–0.96 and 0.86–94 for MSA and county averages, respectively) (Fig. 1, Additional file 1: Figure S3). Compared to measured PM_2.5_, national grid predictions showed consistently lower concentrations in the full range of concentrations, as opposed to census tract predictions which were lower only at the higher concentrations, as many national grid are non-residential locations.

### Life expectancy reanalysis

**Fig. 2:**
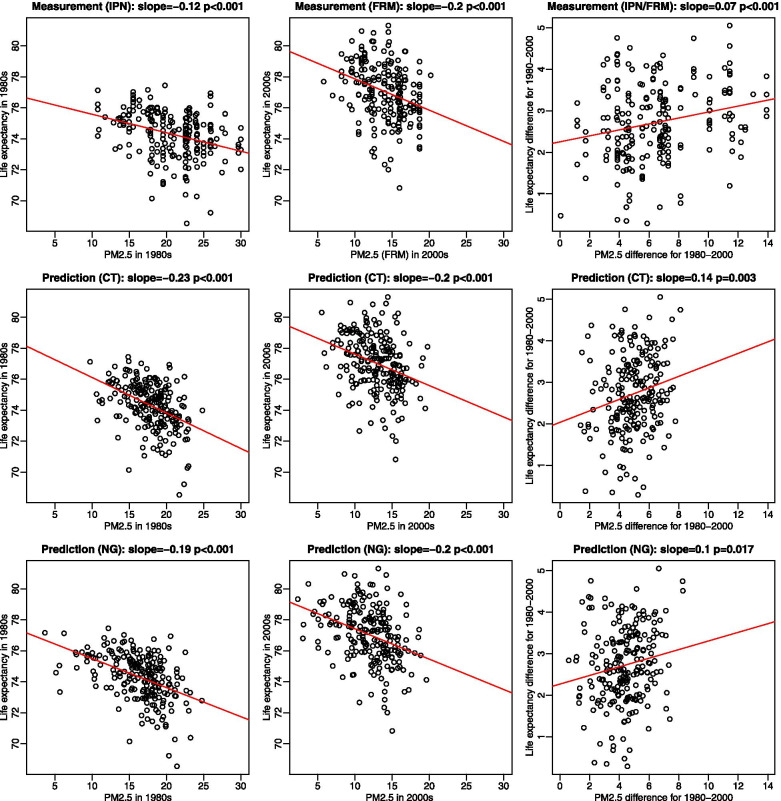
Scatter plots of PM_2.5_ (μg/m^3^) and life expectancy for 1980–2000 across 211 U.S. counties using Metropolitan Statistical Area (MSA) average PM_2.5_ concentrations based on measurements from regulatory monitoring networks (Inhalable Particulate Network (IPN) or Federal Reference Method (FRM)) vs. county average PM_2.5_ concentrations based on predictions at census tract centroids (CT) and national grid coordinates (NG) estimated by the historical prediction model by 1990s, 2000s, and 1980s-2000s

In unadjusted and adjusted models, the association patterns with measured vs. modeled PM_2.5_ were consistent. In the cross-sectional analyses, PM_2.5_ concentrations from monitoring data and historical predictions were negatively associated with life expectancy in each time period, whereas in the change on change analyses, the reduction in PM_2.5_ was positively associated with the increase in life expectancy (Table 2, Fig. 2, Additional file 1: Table S1). In the unadjusted model, the regression coefficients for modeled PM_2.5_ were much higher (1.39 and 1.04 for census tract and national grid predictions, respectively) than measured PM_2.5_ (0.72). Across the four models based on 211 counties, regression coefficients were consistent for measured PM_2.5_ but dramatically dropped for modeled PM_2.5_ particularly in Model 3 when lung cancer/COPD mortality was added. In the primary model (Model 4), with county-specific confounders included, a 10 μg/m^3^ reduction in PM_2.5_ based on census tract predictions was associated with 0.69 year increase in life expectancy (standard error (SE) = 0.31) (Table 2). This estimated improvement in life expectancy was slightly higher and had a larger SE than the estimate reported by Pope et al. (2009) (0.61 years [SE = 0.20]). National grid prediction gave a higher estimate of 0.81 than census tract prediction, with slightly smaller SE (0.29). In the analyses restricted to the 51 counties with the largest population (Models 6 and 7), estimates corresponding to measured PM_2.5_ consistently increased, whereas estimates for modeled PM_2.5_ both decreased and increased. Uncertainty of these estimates increased for modeled PM_2.5_ due to smaller exposure variability, as the smaller variance of predictor variables results in larger standard errors of regression coefficient estimates.

**Table 2:** Estimates of the increase in life expectancy associated with a reduction in PM_2.5_ of 10 μg/m^3^ over approximately 20 years between 1979–1983 and 1999–2000, adjusted for socioeconomic, demographic, and proxy indicators for prevalence of smoking across 211 U.S. counties, using PM_2.5_ concentrations based on Metropolitan Statistical Area (MSA) averages of Inhalable Particulate Network (IPN) or Federal Reference Method (FRM) monitoring data vs. county averages of predictions at census tract centroids and national grid coordinates estimated by the historical prediction model

Model^a^	N of counties	Regression coefficient ± standard error		
		Measured PM_2.5_	Modeled PM_2.5_	
			Census tract	National grid
1	211^b^	0.72 ± 0.29^c^	1.39 ± 0.55^c^	1.04 ± 0.56
2	211	0.83 ± 0.20^c^	1.14 ± 0.49^c^	1.02 ± 0.38^c^
3	211	0.60 ± 0.20^c^	0.54 ± 0.40	0.64 ± 0.31^c^
4	211	0.61 ± 0.20^c^	0.69 ± 0.31^c^	0.81 ± 0.29^c^
5	127	0.55 ± 0.24^c^	0.06 ± 0.41	0.54 ± 0.35
6	51	1.01 ± 0.25^c^	0.16 ± 0.94	0.34 ± 0.64
7	51	0.94 ± 0.23^c^	0.46 ± 0.76	0.63± 0.50

^a﻿^ Model 1: PM_2.5_

Model 2: Model 1 + income + population + 5-yr in-migration + high-school graduates + urban residence + black population + Hispanic population

Model 3: Model 2 + lung-cancer mortality rate + COPD mortality rate

Model 4: Model 1 + income + population + black population + lung-cancer mortality rate + COPD mortality rate

Model 5: Model 4 in 127 counties with the population greater than 100 thousand

Model 6: Model 3 in 51 counties each of which had the large population in each MSA

Model 7: Model 4 in 51 counties each of which had the large population in each MSA

^b^ The same number of counties to that in Pope et al. (2009)’s analysis including some combined counties to have sufficient numbers of deaths for computing life expectancy as described in Pope et al. (2009)

^c^*P* < 0.05

In the sensitivity analysis that replaced county averages of PM_2.5_ with MSA averages in order to compare the same exposure averaging scale as Pope et al. (2009), the patterns were generally consistent, but the significance of the association for census tract predictions in the primary model disappeared. Regression coefficients of both census tract and national grid predictions remained higher with larger SEs than those of measured PM_2.5_ (Additional file 1: Table S2, Additional file 1: Figure S6). The magnitude of estimates increased compared to those of county averages, but their SEs also increased possibly because of reduced variability in MSA-average PM_2.5_, resulting in statistically non-significant effect estimates. Using different years of exposure for averaging, life expectancy estimates were generally larger when PM_2.5_ averages included recent years of predictions between 1999 and 2003 (Additional file 1: Figure S7). However, the 95% confidence intervals were wide and overlapped with the original estimates, indicating that they are not statistically different [22].

## Discussion

This study re-analyzed the change in life expectancy associated with the reduction in PM_2.5_ in the continental U.S. from 1980 to 2010 using modeled exposure estimates. We compared these to the original findings, which were affected by the limited availability of regulatory monitoring data, particularly in 1980s. We hypothesized that predicted exposures, derived from a historical prediction model that was able to extend the spatial and temporal coverage of PM_2.5_ estimates, can provide more accurate health effect estimates and in turn better inform whether decades of improved air quality improved life expectancy. Our estimated improvement in life expectancy attributed to PM_2.5_ reduction was largely consistent with the original analysis estimate.

There have been further studies that attempted to confirm the initial findings in Pope et al. (2009). These studies focused on the assessment of fine-spatial scale exposure at the county level rather than the MSA level and the expansion of counties from 211 to the more than 3,000 counties in the contiguous U.S. [14, 23]. However, their evaluation was limited to the current century and did not incorporate data back in the 1980s and 1990s when major regulatory actions were established and a dramatic change in PM_2.5_ occurred. Using PM_2.5_ regulatory monitoring data in 545 counties for 2000–2007, Correia et al. (2011) found 0.35 year increase in life expectancy for 10 μg/m^3^ reduction in PM_2.5_; these estimates were similar in Bannett et al. (2019) that used predicted PM_2.5_ in 3,082 counties for 1999–2015. For the recent study periods with a slower decline in PM_2.5_ and lower concentrations overall, their estimates of life expectancy improvement were lower compared to ours of 0.67 years. Relying on our validated historical modeling approach, we were able to estimate the change during this critical time period.

The primary life expectancy estimate in our reanalysis using modeled PM_2.5_ was slightly higher and had larger uncertainty than the estimate in the original analysis, although estimates and confidence intervals from both analyses are consistent with increased PM_2.5_ being associated with lower life expectancy. The larger uncertainty can be attributed to reduced variability of our population-representative PM_2.5_ predictions. Reasons for their reduced variability include averaging over 70,000 census tract centroids and the degree of smoothing that is typical of prediction models. Smoothing in prediction may play a significant role particularly in the 1980s when spatially extensive monitoring data did not exist. The larger variability of measured PM_2.5_ could be due to some monitoring sites located at extremely polluted and/or unpolluted locations as well as those IPN sites that operated in only a few seasons. Our previous study showed that 102 IPN sites for 1979–1982 decreased to 16 for 1980–1981, when the site inclusion criteria for computing representative annual average concentrations of PM_2.5_ were applied [15]. Changes over time in locations of monitoring sites and different sampling schedules among monitors might also have affected the variability in the originally reported PM_2.5_ difference. We note that exposure measurement error driven by these spatial and/or temporal misalignments could lead to biases in either direction, as well as incorrect standard errors of health effect estimates [24–26]. In addition, when we replaced our county-average predictions with MSA-average predictions in our sensitivity analysis to match to the exposure averaging scale of the original analysis, standard errors of estimates increased and the primary model estimate became statistically non-significant. As opposed to limited monitoring data in 1980s, our prediction model allowed us to estimate county averages, resulting in good alignment of the exposure and the outcome and increased exposure variability.

By expanding the temporal and spatial scales of PM_2.5_ to cover the entire continental U.S. back to 1980, our study provides a mechanism for future high-quality policy-relevant analyses of PM_2.5_ health impacts. Mortality and morbidity data are often available much earlier than the establishment of the extensive spatial monitoring of PM_2.5_. Our PM_2.5_ estimates linked to administrative health data allow the assessment of the health benefits achieved from the reduction of PM_2.5_ over time to be evaluated [27]. In addition, areas with no nearby regulatory monitoring sites have been shown to have different demographic characteristics than areas represented by monitors [28]. It may be inadequate to rely on simpler area averages computed directly from regulatory monitoring sites to capture the differences in susceptibility depending on the sociodemographic subgroup. For example, only 567 (18%) of the 3,109 counties in the continental U.S. in 2000 had at least one regulatory monitor with sufficient daily measurements to provide representative annual averages (Additional file 1: Figure S8). The median number of monitoring sites per MSA based on the 2000 Census was 1 (interquartile-range: 1 – 3), with median area and population per monitoring site of 2,232 km^2^ and 198,306 people.

The higher and less uncertain estimates of the PM_2.5_ effect on life expectancy when PM_2.5_ was obtained from national grid-based predictions, in comparison to census tract-based predictions, could be due to exposure measurement error because grid locations do not adequately represent locations where people live. National grid coordinates possibly include many non-residential locations with low concentrations in addition to residential locations, whereas most census tract centroids are likely to fall in residential areas and better represent residential exposure. On average, the grid-based estimates underestimated area-average PM_2.5_ concentrations compared to the census tract-based estimates.

One limitation of this study is that we restricted the life expectancy analysis study period to 1980–2000 to facilitate comparison with the results in the original analysis. Future analyses could leverage updated life expectancy data to investigate other time periods including more recent time periods and ascertain whether the results are consistent. Furthermore, the two estimates of PM_2.5_ reduction from monitored vs. modeled PM_2.5_ on the improvement in life expectancy are likely to have different measurement error impacts. Future studies also should quantify these differences.

## Conclusions

Using modeled PM_2.5_ concentrations expanded spatially and temporally to overcome limited coverage of monitoring data, we replicated Pope et al.’s life expectancy analysis, work that has been influential in environmental policy and regulatory accountability. Our analysis confirmed the previous findings of the contribution of improved air quality to improved life expectancy.

## Supplementary Information

**Additional file 1: Table S1**. Estimates of life expectancy associated with a 10 μg/m^3^ increase in long-term average PM_2.5_ concentrations by the two time periods (1979-1983 and 1999-2000), adjusted for socioeconomic, demographic, and proxy indicators for prevalence of smoking across 211 U.S. counties, using PM_2.5_ concentrations based on Metropolitan Statistical Area (MSA) averages of Inhalable Particulate Network (IPN) or Federal Reference Method (FRM) monitoring data vs. county averages of predictions at census tract centroids and national grid coordinates estimated by the historical prediction model. **Table S2**. Estimates of the increase in life expectancy associated with a reduction in PM_2.5_ of 10 μg/m^3^ over approximately 20 years between 1979-1983 and 1999-2000, adjusted for socioeconomic, demographic, and proxy indicators for prevalence of smoking in 211 U.S. counties, using Metropolitan Statistical Area (MSA) average PM_2.5_ concentrations based on measurements from regulatory monitoring networks (Inhalable Particulate Network (IPN) or Federal Reference Method (FRM)) vs. predictions at census tract centroids and national grid coordinates estimated by the historical prediction model. **Figure S1**. Maps of 3,109 counties in the continental U.S., and 25-km grid coordinates, regulatory monitoring sites, and census tract centroids in Los Angeles county in 2010. **Figure S2**. Map of the 211 U.S. counties included in the life expectancy analysis in Pope et al. 2009. **Figure S3**. Scatter plots of PM_2.5_ concentrations using Metropolitan Statistical Area (MSA) averages based on measurements from regulatory monitoring networks (Inhalable Particulate Network (IPN) or Federal Reference Method (FRM)) vs. county averages based on predictions at census tract centroids (CT) and national grid coordinates (NG) estimated by the historical prediction model by 1980s (1979-1983), 2000s (1999-2000), and 1980s-2000s (1979-1983 minus 1999-2000) (green and red lines for the identity and best-fitted lines, respectively). **Figure S4**. Scatter plots of county-level annual averages of PM_2.5_ (μg/m^3^) predictions at census tract centroids estimated by the historical exposure prediction model against Metropolitan Statistical Area (MSA) averages of PM_2.5_ measurements from regulatory monitoring networks (Inhalable Particulate Network (IPN) or Federal Reference Method (FRM)) by 1980s (1979-1983), 2000s (1999-2000), and 1980s-2000s (1979-1983 minus 1999-2000) (same plots to those in the first column of Figure S3 but with colored dots indicating the rank of the concentration in 1980s). **Figure S5**. Maps of county-level annual-average PM_2.5_ concentrations (μg/m3) in 1980, 1990, 2000, and 2010 based on census tract centroid predictions estimated by the historical prediction model. **Figure S6**. Scatter plots of PM_2.5_ (μg/m^3^) and life expectancy for 1980-2000 between 1979-1983 and 1999-2000 across 211 U.S. counties using Metropolitan Statistical Area (MSA) average PM_2.5_ concentrations based on measurements from regulatory monitoring networks (Inhalable Particulate Network (IPN) or Federal Reference Method (FRM)) vs. predictions at census tract centroids (CT) and national grid coordinates (NG) estimated by the historical prediction model by 1990s, 2000s, and 1980s-2000s. **Figure S7**. Effect estimates and 95% confidence intervals of the life expectancy increase for a PM_2.5_ reduction of 10 μg/m^3^ between 1980s and 2000s across 211 U.S. counties, adjusted for socioeconomic, demographic, and proxy indicators for prevalence of smoking in four health analysis models, by different years of PM_2.5_ predictions at census tract centroids estimated by the historical prediction model (black closed circles indicating effect estimates of PM_2.5_ reduction based on IPN and FRM measurements between 1979-1983 and 1999-2000 in Pope et al. (2009); black open circles based on predictions for the same averaging periods as Pope et al. (2009) in our re-analysis; all the other symbols and colors based on predictions using different single start and end years). **Figure S8**. Map of 567 counties where there is at least one regulatory monitoring site after applying the minimum inclusion criteria for computing annual averages in 2000.

## Data Availability

The datasets analyzed in this study are available from the corresponding author SY Kim (sykim@ncc.re.kr) after coauthors’ approval of the request.

## Abbreviations

COPD: Chronic obstructive pulmonary disease
FRM: Federal Reference Methods
IPN: Inhalable Particulate Network
IMPROVE: Interagency Monitoring of Protected Visual Environments
MSA: Metropolitan Statistical Area
MESA Air: Multi-ethnic Study of Atherosclerosis and Air Pollution
PLS: Partial least squares
PM_2.5_: Particulate matter with a diameter equal to or less than 2.5 μm
SD: Standard deviation
SE: Standard error
