# Supplementary material for: Reanalysis of the association between reduction in long-term PM2.5 concentrations and improved life expectancy
Source: Environ Health. 2021 Sep 13;20:102. doi: 10.1186/s12940-021-00785-0 (PMC8439090; doi:10.1186/s12940-021-00785-0)
Supplement: Supplementary file 1 — Additional file 1: Table S1. Estimates of life expectancy associated with a 10 μg/m3 increase in long-term average PM2.5 concentrations by the two time periods (1979-1983 and 1999-2000), adjusted for socioeconomic, demographic, and proxy indicators for prevalence of smoking across 211 U.S. counties, using PM2.5 concentrations based on Metropolitan Statistical Area (MSA) averages of Inhalable Particulate Network (IPN) or Federal Reference Method (FRM) monitoring data vs. county averages of predictions at census tract centroids and national grid coordinates estimated by the historical prediction model. Table S2. Estimates of the increase in life expectancy associated with a reduction in PM2.5 of 10 μg/m3 over approximately 20 years between 1979-1983 and 1999-2000, adjusted for socioeconomic, demographic, and proxy indicators for prevalence of smoking in 211 U.S. counties, using Metropolitan Statistical Area (MSA) average PM2.5 concentrations based on measurements from regulatory monitoring networks (Inhalable Particulate Network (IPN) or Federal Reference Method (FRM)) vs. predictions at census tract centroids and national grid coordinates estimated by the historical prediction model. Figure S1. Maps of 3,109 counties in the continental U.S., and 25-km grid coordinates, regulatory monitoring sites, and census tract centroids in Los Angeles county in 2010. Figure S2. Map of the 211 U.S. counties included in the life expectancy analysis in Pope et al. 2009. Figure S3. Scatter plots of PM2.5 concentrations using Metropolitan Statistical Area (MSA) averages based on measurements from regulatory monitoring networks (Inhalable Particulate Network (IPN) or Federal Reference Method (FRM)) vs. county averages based on predictions at census tract centroids (CT) and national grid coordinates (NG) estimated by the historical prediction model by 1980s (1979-1983), 2000s (1999-2000), and 1980s-2000s (1979-1983 minus 1999-2000) (green and red lines for the identity and best-fitted l [file 12940_2021_785_MOESM1_ESM.docx]

Supplemental materials

Reanalysis of the association between reduction in long-term PM_2.5_ concentrations and improved life expectancy

Sun-Young Kim, C. Arden Pope III, Julian D. Marshall, Neal Fann, Lianne Sheppard

Table S1. Estimates of life expectancy associated with a 10 μg/m^3^ increase in long-term average PM_2.5_ concentrations by the two time periods (1979-1983 and 1999-2000), adjusted for socioeconomic, demographic, and proxy indicators for prevalence of smoking across 211 U.S. counties, using PM_2.5_ concentrations based on Metropolitan Statistical Area (MSA) averages of Inhalable Particulate Network (IPN) or Federal Reference Method (FRM) monitoring data vs. county averages of predictions at census tract centroids and national grid coordinates estimated by the historical prediction model

| Period | Model^1^ | N of counties | Regression coefficient ± standard error | | |
| --- | --- | --- | --- | --- | --- |
|  |  |  | Measured PM_2.5_ | Modeled PM_2.5_ | |
|  |  |  |  | Census tract | National grid |
| 1979-1983 | 1 | 211 | -1.19 ±0.27 | -2.29±0.36 | -1.88±0.25 |
|  | 2 | 211 | -0.76 ±0.29 | -1.79±0.41 | -1.35±0.28 |
|  | 3 | 211 | -0.78±0.29 | -1.82±0.40 | -1.35±0.28 |
|  | 4 | 211 | -1.21±0.27 | -2.81±0.35 | -2.13±0.27 |
|  | 5 | 127 | -1.16±0.26 | -3.26±0.50 | -2.11±0.29 |
|  | 6 | 51 | -1.05±0.38 | -1.73±0.66 | -1.44±0.42 |
|  | 7 | 51 | -1.48±0.37 | -2.66±0.49 | -1.93±0.33 |
| 1999-2000 | 1 | 211 | -2.02±0.50 | -2.03±0.50 | -1.96±0.36 |
|  | 2 | 211 | -1.03±0.57 | -1.27±0.53 | -1.21±0.43 |
|  | 3 | 211 | -0.95±0.57 | -1.23±0.51 | -1.17±0.41 |
|  | 4 | 211 | -1.80±0.44 | -2.24±0.43 | -2.00±0.34 |
|  | 5 | 127 | -1.72±0.51 | -2.38±0.50 | -2.02±0.40 |
|  | 6 | 51 | -1.55±0.68 | -1.45±0.71 | -1.56±0.53 |
|  | 7 | 51 | -2.24±0.57 | -2.31±0.55 | -2.07±0.43 |

1) Model 1: PM_2.5_

Model 2: Model 1 + income + population + 5-yr in-migration + high-school graduates + urban residence + black population + Hispanic population

Model 3: Model 2 + lung-cancer mortality rate + COPD mortality rate

Model 4: Model 1 + income + population + black population + lung-cancer mortality rate + COPD mortality rate

Model 5: Model 4 in 127 counties with the population greater than 100 thousand

Model 6: Model 3 in 51 counties each of which had the largest population in each MSA

Model 7: Model 4 in 51 counties each of which had the largest population in each MSA

Table S2. Estimates of the increase in life expectancy associated with a reduction in PM_2.5_ of 10 μg/m^3^ over approximately 20 years between 1979-1983 and 1999-2000, adjusted for socioeconomic, demographic, and proxy indicators for prevalence of smoking in 211 U.S. counties, using Metropolitan Statistical Area (MSA) average PM_2.5_ concentrations based on measurements from regulatory monitoring networks (Inhalable Particulate Network (IPN) or Federal Reference Method (FRM)) vs. predictions at census tract centroids and national grid coordinates estimated by the historical prediction model

| Model^1^ | N of counties | Regression coefficient ± standard error | | |
| --- | --- | --- | --- | --- |
|  |  | Measured PM_2.5_ | Modeled PM_2.5_ | |
|  |  |  | Census tract | National grid |
| 1 | 211 | 0.72 ± 0.29^2^ | 1.75 ± 0.74^2^ | 1.42 ± 0.67^2^ |
| 2 | 211 | 0.83 ± 0.20^2^ | 1.37 ± 0.51^2^ | 1.44 ± 0.47^2^ |
| 3 | 211 | 0.60 ± 0.20^2^ | 0.69 ± 0.39 | 0.96 ± 0.35^2^ |
| 4 | 211 | 0.61 ± 0.20^2^ | 0.67 ± 0.39 | 0.97 ± 0.38^2^ |
| 5 | 127 | 0.55 ± 0.24^2^ | 0.09 ± 0.46 | 0.82 ± 0.45 |
| 6 | 51 | 1.01 ± 0.25^2^ | 0.41 ± 0.92 | 0.90 ± 0.73 |
| 7 | 51 | 0.94 ± 0.23^2^ | 0.78 ± 0.84 | 1.15 ± 0.58 |

1) Model 1: PM_2.5_

Model 2: Model 1 + income + population + 5-yr in-migration + high-school graduates + urban residence + black population + Hispanic population

Model 3: Model 2 + lung-cancer mortality rate + COPD mortality rate

Model 4: Model 1 + income + population + black population + lung-cancer mortality rate + COPD mortality rate

Model 5: Model 4 in 127 counties with the population greater than 100 thousand

Model 6: Model 3 in 51 counties each of which had the largest population in each MSA

Model 7: Model 4 in 51 counties each of which had the largest population in each MSA

2) P < 0.05


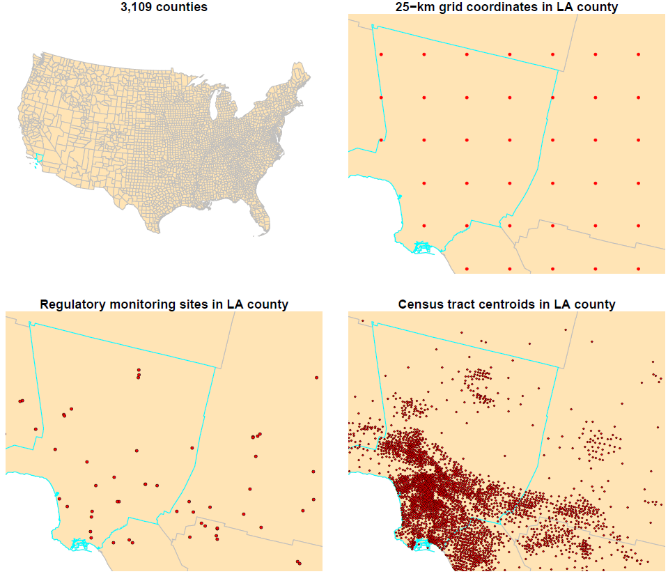


Figure S1. Maps of 3,109 counties in the continental U.S., and 25-km grid coordinates, regulatory monitoring sites, and census tract centroids in Los Angeles county in 2010


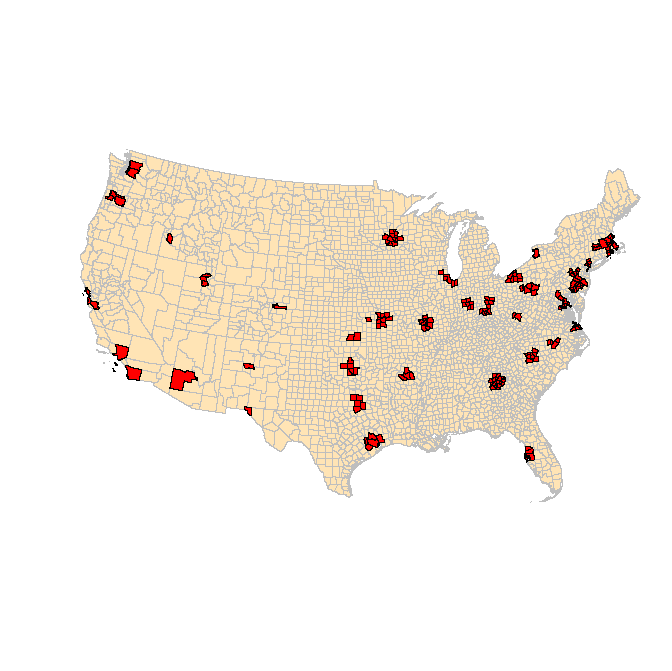


Figure S2. Map of the 211 U.S. counties included in the life expectancy analysis in Pope et al. 2009


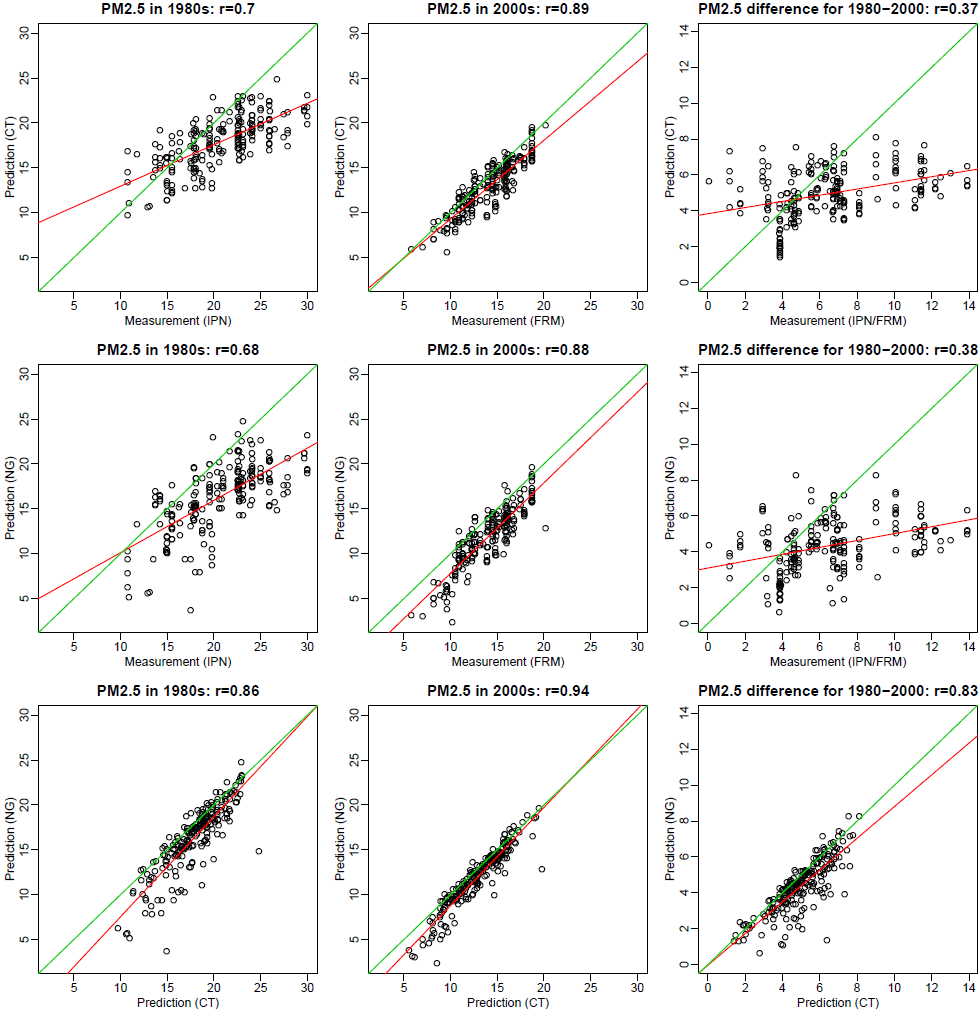


Figure S3. Scatter plots of PM_2.5_ concentrations using Metropolitan Statistical Area (MSA) averages based on measurements from regulatory monitoring networks (Inhalable Particulate Network (IPN) or Federal Reference Method (FRM)) vs. county averages based on predictions at census tract centroids (CT) and national grid coordinates (NG) estimated by the historical prediction model by 1980s (1979-1983), 2000s (1999-2000), and 1980s-2000s (1979-1983 minus 1999-2000) (green and red lines for the identity and best-fitted lines, respectively)


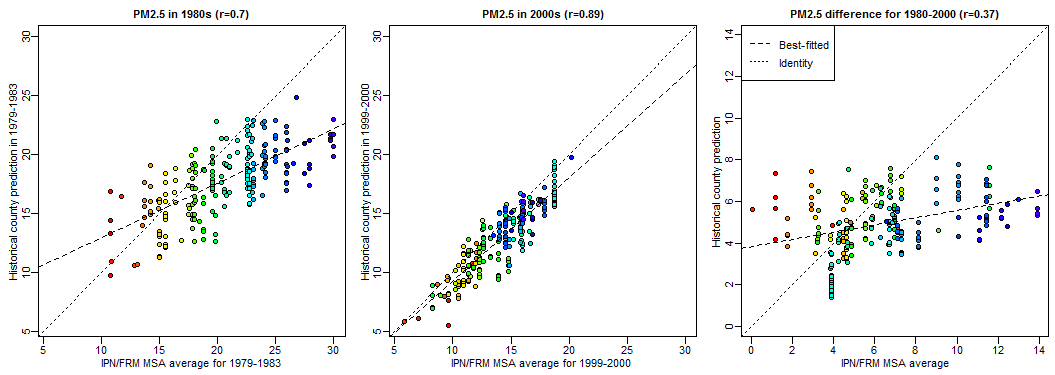


Figure S4. Scatter plots of county-level annual averages of PM_2.5_ (μg/m^3^) predictions at census tract centroids estimated by the historical exposure prediction model against Metropolitan Statistical Area (MSA) averages of PM_2.5_ measurements from regulatory monitoring networks (Inhalable Particulate Network (IPN) or Federal Reference Method (FRM)) by 1980s (1979-1983), 2000s (1999-2000), and 1980s-2000s (1979-1983 minus 1999-2000) (same plots to those in the first column of Figure S3 but with colored dots indicating the rank of the concentration in 1980s)


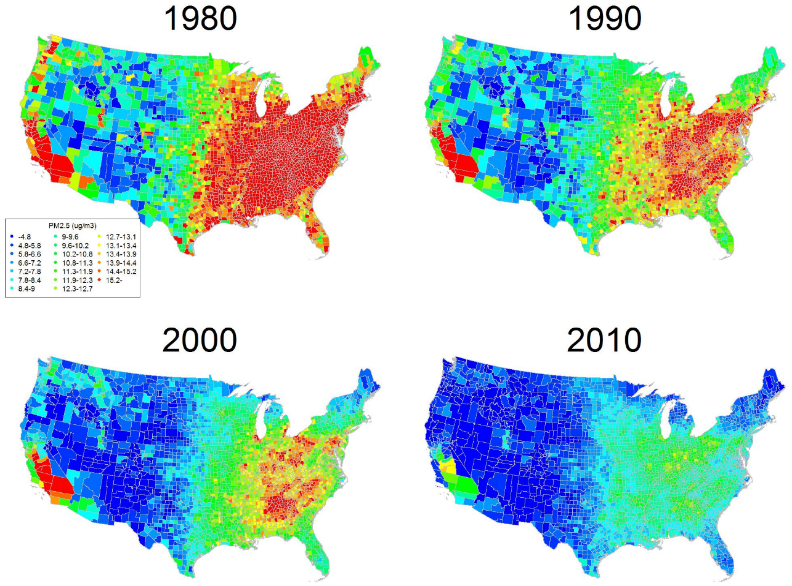


Figure S5. Maps of county-level annual-average PM_2.5_ concentrations (μg/m3) in 1980, 1990, 2000, and 2010 based on census tract centroid predictions estimated by the historical prediction model


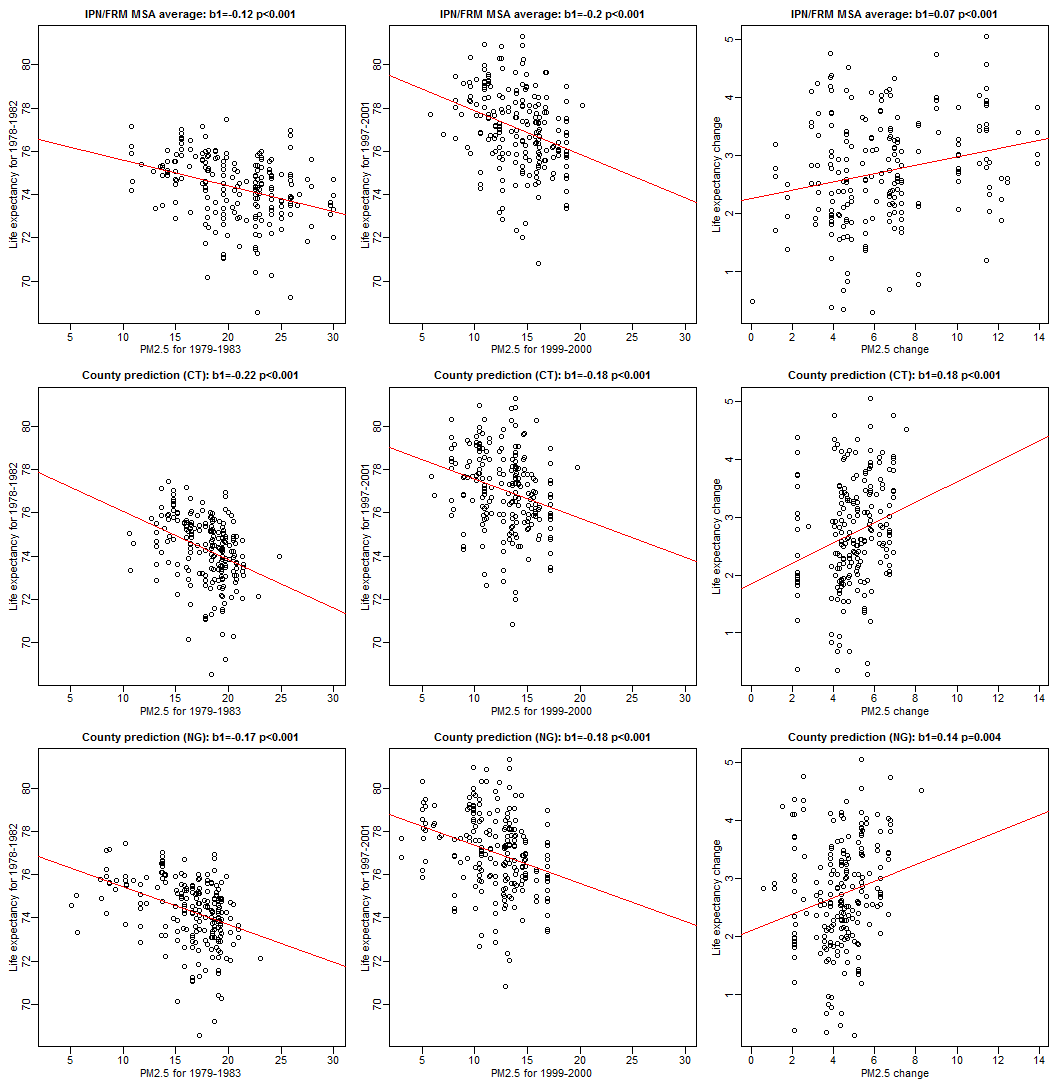


Figure S6. Scatter plots of PM_2.5_ (μg/m^3^) and life expectancy for 1980-2000 between 1979-1983 and 1999-2000 across 211 U.S. counties using Metropolitan Statistical Area (MSA) average PM_2.5_ concentrations based on measurements from regulatory monitoring networks (Inhalable Particulate Network (IPN) or Federal Reference Method (FRM)) vs. predictions at census tract centroids (CT) and national grid coordinates (NG) estimated by the historical prediction model by 1990s, 2000s, and 1980s-2000s


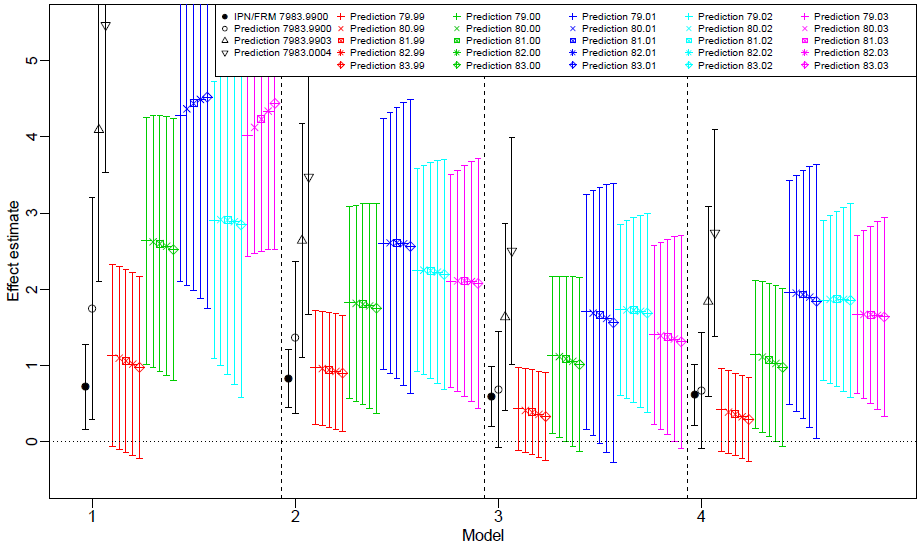


Figure S7. Effect estimates and 95% confidence intervals of the life expectancy increase for a PM_2.5_ reduction of 10 μg/m^3^ between 1980s and 2000s across 211 U.S. counties, adjusted for socioeconomic, demographic, and proxy indicators for prevalence of smoking in four health analysis models, by different years of PM_2.5_ predictions at census tract centroids estimated by the historical prediction model (black closed circles indicating effect estimates of PM_2.5_ reduction based on IPN and FRM measurements between 1979-1983 and 1999-2000 in Pope et al. (2009); black open circles based on predictions for the same averaging periods as Pope et al. (2009) in our re-analysis; all the other symbols and colors based on predictions using different single start and end years)


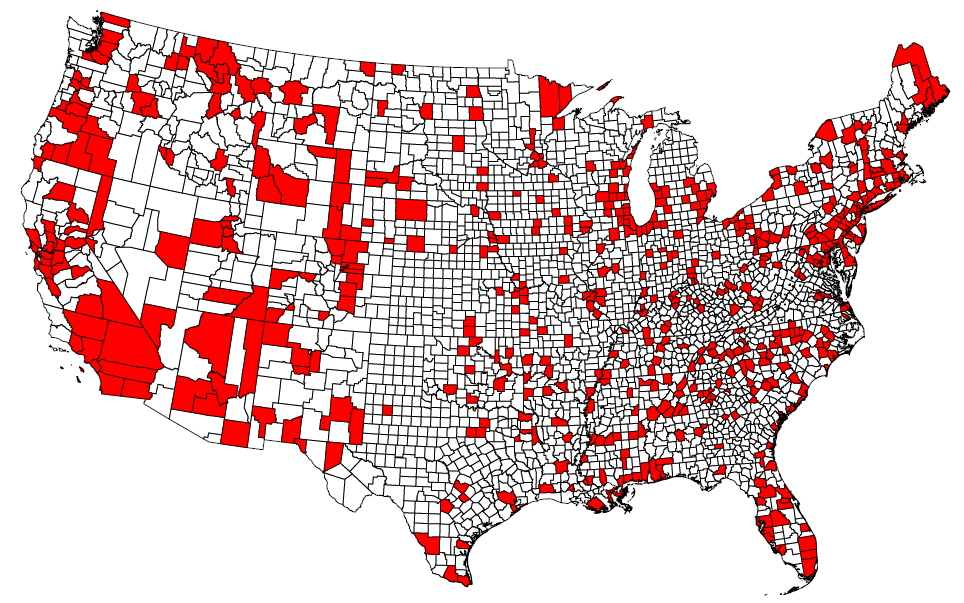


Figure S8. Map of 567 counties where there is at least one regulatory monitoring site after applying the minimum inclusion criteria for computing annual averages in 2000
